# A new FRAX model for Brazil

**DOI:** 10.1007/s11657-023-01354-3

**Published:** 2023-11-28

**Authors:** B. H. Albergaria, C. A. F. Zerbini, M. Lazaretti-Castro, S. R. Eis, T. Vilaca, H. Johansson, N. C. Harvey, E. Liu, L. Vandenput, M. Lorentzon, M. Schini, E. McCloskey, J. A. Kanis

**Affiliations:** 1 Osteoporosis Research and Diagnosis Center - CEDOES, Vitoria, Brazil; 2 Federal University of Espirito Santo, Vitoria, Brazil; 3 Centro Paulista de Investigação Clinica, Sao Paulo, Brazil; 4 Federal University of São Paulo, Sao Paulo, Brazil; 5 Mellanby Centre for Musculoskeletal Research, Department of Oncology and Metabolism, University of Sheffield, Sheffield, UK; 6 Mary McKillop Institute for Health Research, Australian Catholic University, Melbourne, Australia; 7 Centre for Metabolic Bone Diseases, University of Sheffield, Sheffield, UK; 8 Sahlgrenska Osteoporosis Centre, Institute of Medicine, University of Gothenburg, Gothenburg, Sweden; 9 MRC Lifecourse Epidemiology Centre, University of Southampton, Southampton, UK; 10 NIHR Southampton Biomedical Research Centre, University of Southampton and University Hospital Southampton NHS Foundation Trust, Southampton, UK

**Keywords:** FRAX, Fracture, Fracture probability, Epidemiology, Hip fracture

## Abstract

***Summary*:**

Fracture probabilities derived from the original FRAX model for Brazil were compared to those from an updated model based on more recent regional estimates of the incidence of hip fracture. Fracture probabilities were consistently lower in the updated FRAX model. Despite large differences between models, differences in the rank order of fracture probabilities were minimal.

**Objective:**

Recent epidemiological data indicate that the risk of hip fracture in Brazil is lower than that used to create the original FRAX model. This paper describes the epidemiology of hip fracture in Brazil and the synthesis of an updated FRAX model with the aim of comparing this new model with the original model.

**Methods:**

Hip fracture rates from three cities in three regions were combined, weighted by the population of each region. For other major fractures, incidence rates for Brazil were estimated using Swedish ratios for hip to other major osteoporotic fracture (humerus, forearm or clinical vertebral fractures). Mortality estimates were taken from the UN.

**Results:**

Compared to the original FRAX model, the updated model gave lower 10-year fracture probabilities in men and women at all ages. Notwithstanding, there was a very close correlation in fracture probabilities between the original and updated models (*r* > 0.99) so that the revisions had little impact on the rank order of risk.

**Conclusion:**

The disparities between the original and updated FRAX models indicate the importance of updating country-specific FRAX models with the advent of significant changes in fracture epidemiology.

## Introduction

FRAX® is a computer-based algorithm (http://www.shef.ac.uk/FRAX) developed by the then World Health Organization Collaborating Centre for Metabolic Bone Diseases and first released in 2008. The algorithm, intended primarily for use in primary care, calculates fracture probability from easily obtained clinical risk factors (CRFs) in men and women [1, 2]. The output of FRAX is the 10-year probability of a major fracture (hip, clinical spine, humerus or wrist fracture) and the 10-year probability of hip fracture. Probability is calculated from the risk of fracture and death according to age, body mass index (BMI) and dichotomized risk factors comprising prior fragility fracture, parental history of hip fracture, current tobacco smoking, long-term oral glucocorticoid use, rheumatoid arthritis, other causes of secondary osteoporosis and excessive alcohol consumption. Femoral neck bone mineral density (BMD) can be optionally input to enhance fracture risk prediction [3].

The risk of hip fracture and probably of other osteoporotic fractures varies markedly around the world. In addition, the age-specific risk of death varies between countries. This variation also contributes to the heterogeneity in fracture probability [4]. For this reason, FRAX models are calibrated to those countries where the epidemiology of fracture and death is known. Models are currently available for 81 countries or territories covering more than 80% of the world population age 50 years or more [5].

A FRAX model for Brazil was released on May 1, 2013 (http://www.shef.ac.uk/FRAX). FRAX Brazil relied on data now more than 20 years old [6]. Age- and sex-stratified hip fracture incidence rates were extracted from four regional estimates from the age of 40 years [7–10]. The estimates were from Porto Allegre, Marilia, Sobral and Fortaleza. For other major fractures, incidence rates for Brazil were estimated using Swedish ratios for hip to other major osteoporotic fracture (humerus, forearm or clinical vertebral fractures). Since then, several more recent studies on the epidemiology of fractures have been published [11, 12] including the Brazilian Validation Osteoporosis Study (BRAVOS), specifically conducted to update FRAX [13]. These more recent publications indicate that hip fracture incidence in recent years is substantially lower than that used for the original version of FRAX.

The aim of the present study was to provide an update of the FRAX model for Brazil using the data from the BRAVOS study and to compare probabilities with the original FRAX model.

## Methods

The BRAVOS study documented the incidence of hip fracture in three representative Brazilian cities, Belem (in the Northeast region) Vitoria (Southeast) and Joinville (South). Details have been previously published [13]. In brief, this was a retrospective, observational study including all patients aged ≥ 50 years admitted in hospitals because of a hip fracture from 2010 to 2012. Data were obtained from medical records. Fractures (ICD-10 codes S72.0, S72.1, S72.2) were extracted by trained personnel and a central review process was established to confirm the diagnosis of hip fractures and the completeness and accuracy of the data. If the patient sustained a contralateral hip fracture during the survey, it was registered as a new fracture. Annual incidence of hip fracture was determined in men and in women in 5-year age intervals rates. Age-standardised hip fracture rates were lowest in Belem, intermediate in Vitoria and highest in Joinville. The hip fracture incidence in Belem was significantly lower than that in Vitoria and Joinville.

Brazil is divided in five regions, but the majority of the population lives in the Southeast (42%), Northeast (28%) and South (14%). In order to create a single FRAX model, hip fracture rates of the three cities from these regions were combined weighted by the population of each region. This assumes that the hip fracture incidence in each city was representative of each region. The point estimates of incidence at each age interval were log-transformed and, since the data followed different linear trends over different ages, piecewise linear regression was used to more accurately summarise the relationship between age and log incidence. Breakpoints for the regression were at 62 and 87 years of age for men and 62 and 82 years for women. The estimated number of hip fractures in men and women nationwide was calculated from the age of 50 years from population demography [14]. The incidence of other major osteoporotic fractures (clinical spine, distal forearm and proximal humerus) could not be determined from literature sources. It was assumed, therefore, that these age- and gender-specific ratios found in Sweden were comparable to those in Brazil. This assumption has also been used for many of the FRAX models with incomplete epidemiological information. Available information suggests that the age- and gender-stratified pattern of fracture is very similar in the Western world and Australia [15–19].

The development and validation of FRAX has been extensively described [1, 2]. The risk factors used were based on a systematic set of meta-analyses of population-based cohorts worldwide and validated in independent cohorts with over a million patient-years of follow-up. The construct of the FRAX model for Brazil required the beta coefficients of the risk factors in the original FRAX model and the incidence rates of hip fracture and mortality rates for Brazil [6]. The relative importance of the beta coefficients for death and fracture was assumed to be similar in Brazil, as has been shown across many countries [2]. However, absolute age-specific fracture risk and mortality rates differ from country to country [4]. Consequently, for each age category, the hazard function was calibrated to match the mean risk (both fracture risk and mortality rate) for that specific age group in Brazil, without altering the relative importance of the beta coefficients. National mortality rates used data for 2015–2019 available from the United Nations [20].

### Comparison of models

For the purpose of comparing the new FRAX model and the original model, the probabilities of a major osteoporotic fracture (hip, clinical spine, forearm and humeral fractures) and of hip fracture alone were computed in men and women at ages 50, 60, 70 and 80 years for all possible combinations of clinical risk factors at BMD *T*-scores between 0 and − 3.5 SD in 0.5 SD steps with a BMI set to 25 kg/m^2^ [19, 21]. Thus, we considered all combinations of six risk factors and eight values of BMD giving a total number of combinations of 512 for each age. The probabilities were calculated with the FRAX desktop multi-patient entry tool (FRAX Desktop (frax-tool.org)). Note that this was not a population simulation, but an array of all possible combinations. The relationship between probabilities of the original and the updated FRAX model followed different linear trends over different probability estimates so that piecewise linear regression was used to more accurately summarise the relationship between the FRAX models. The correlation between the probabilities derived from the original and updated models used a breakpoint at 30% for the probabilities of a major osteoporotic fracture and hip fracture. Tabular data were used to compare probabilities between the two versions at the 50th (median) percentile of the distribution of the surrogate model. Differences in the authentic model from the surrogate model at these percentiles were expressed as 95% tolerance intervals (TI).

## Results

**Fig. 1:**
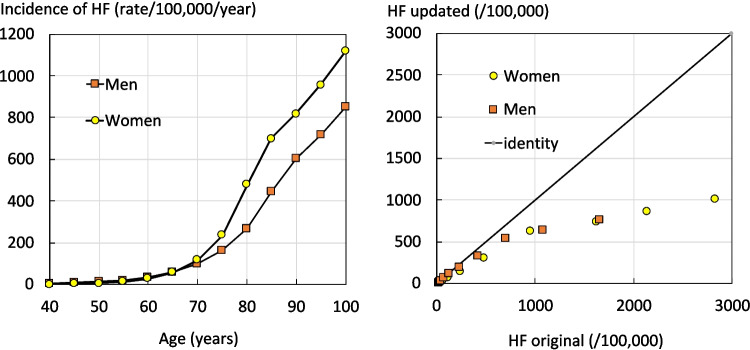
The annual incidence of hip fractures (HF; rate/100,000) by age and sex in Brazil among men (square symbols) and women (circles). The right-hand panel compares the updated incidence used in the present study with that used for the original FRAX model

The incidence of hip fractures increased exponentially with age and women had a risk about 50% higher than that of men from the age of 70 years (Fig. 1). Hip fracture rates were approximately half of than those used in the original FRAX model. The number of hip fractures nationally in 2021 was estimated at 56,526, of which 19,555 (35%) were in men and 36,971 in women.

**Fig. 2:**
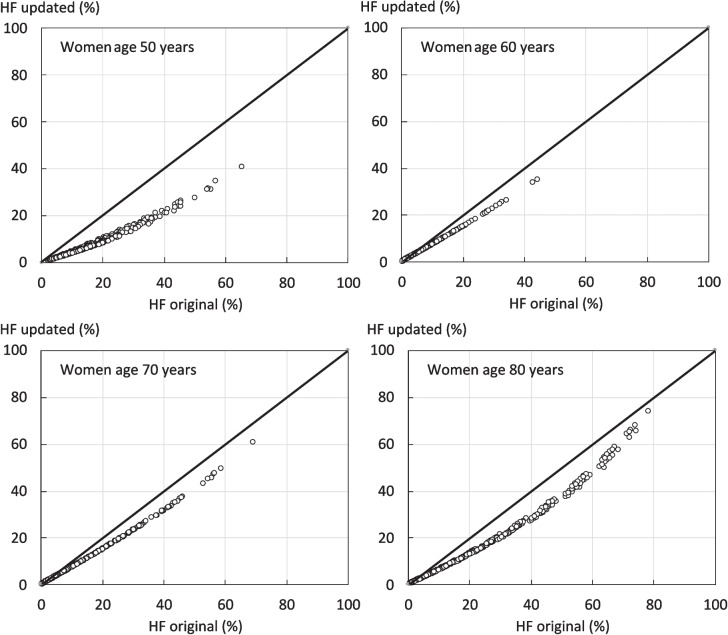
Comparison of 10-year probability of a hip fracture (HF) using the original FRAX tool for the Brazilian female population and the update tool for multiple clinical scenarios. The diagonal line shows the line of identity

The relationship between the probabilities of a hip fracture derived from all permutations of risk factor and age combinations in the two versions of FRAX is shown for women age 50 to 80 years in Fig. 2. At all ages, there was a close correlation between the two estimates (*r* > 0.99). The update version gave consistently lower probabilities than the original model at all ages. The effect in men was very similar to that of women.

Table 1 provides median values for the 10-year probability of hip fracture and MOF for the original and update FRAX model. The median value for hip fracture probability was lower by 26–44% in both men and women depending on age. In the case of MOF, there was also a close correlation between the two estimates (*r* > 0.99) at all ages. The update version gave lower estimates for MOF probability than the original model by 20–56% in both men and women depending on age.

**Table 1:** 10-year probability (%) of a major osteoporotic fracture (MOF) or a hip fracture with 95% tolerance intervals (TI) in men and women at the median of the probability distribution (original version) by age.

Age	Men				Women			
	Original	Update			Original	Update		
	Median		95% TI	*r* value	Median		95% TI	*r* value
MOF								
50	10.4	4.8	3.7–5.8	0.992	10.9	4.8	3.8–5.8	0.991
60	9.6	6.8	6.4–7.32	0.999	11.9	8.4	8.0–8.8	0.999
70	10.2	8.2	7.8–8.5	0.999	13.2	10.6	10.0–11.1	0.999
80	14.3	9.5	8.5–10.4	0.995	21.0	12.7	10.6–14.8	0.991
Hip fracture								
50	2.5	1.4	1.2–1.6	0.998	1.6	0.9	0.7–1.0	0.999
60	2.7	2.0	1.8–2.2	1.000	2.1	1.5	1.4–1.7	1.000
70	5.7	4.2	3.9–4.5	0.999	5.4	4.0	3.6–4.3	0.999
80	11.0	7.5	6.7–8.3	0.996	13.6	8.6	6.8–10.3	0.992

**Fig. 3:**
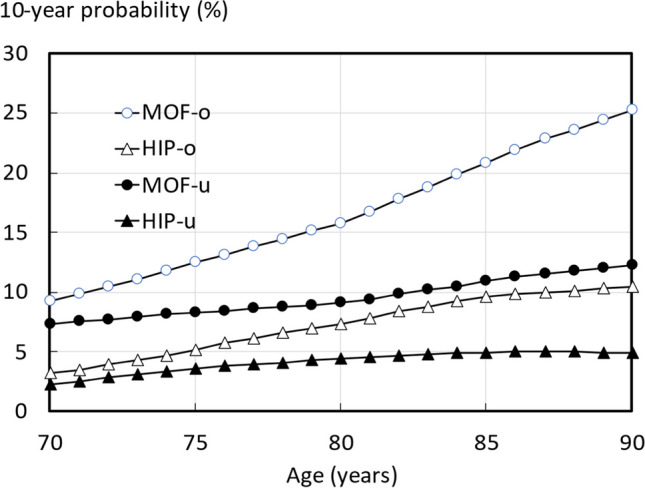
10-year probabilities (%) of a major osteoporotic fracture (MOF) and hip fracture (HIP) in women with a prior fracture and a body mass index of 25 kg/m^2^ using the original FRAX model (MOF-o and HIP-o) and the updated model (MOF-u and HIP-u)

Examples of fracture probabilities with the original and updated model in a woman with a prior fracture and a body mass index of 25kg/m^2^ is shown in Fig. 3.

## Discussion

In this study, we used recent data on the incidence of hip fracture in Brazil to update the original FRAX model [6]. In brief, the revision provided lower estimates of fracture probability at all ages than the original model. Importantly, the update had little impact on the categorisation of risk, since the rank order of fracture probability did not change. In the clinical scenarios presented in this paper, the correlation coefficients between the original and revised versions for fracture probability exceeded 0.99, so that the one can be accurately predicted from the other. In other words, an individual at the 90th percentile of risk would still be at the 90th percentile of risk using the updated FRAX tool. Thus, the consequences of improving accuracy reside in the absolute number generated and not in the rank order of risk within a population. Similar close correlations between original and revised FRAX models have been reported for the USA, Armenia and Ecuador [19, 21, 22]. Because of the close correlation, the difference in probabilities is of little consequence to the management of patients. There is a useful analogy with the different DXA devices available, where a substantial difference in femoral neck BMD is seen between Hologic and Lunar machines, but the *T*-score derived from these is more or less identical [23]. However, marked difficulties arise when fracture probabilities are used in health economic analysis to inform practice guidelines or to set probability-based intervention thresholds. In this context, accuracy is mandatory.

The principal reason that the revised model gave lower fracture probabilities than the original model for Brazil lay in the lower incidence of hip fracture in the BRAVOS study. That hip fracture risk is lower than that utilised for the original model is consistent with other more recent data. Thus, a similarly low incidence was determined from a retrospective analysis of the Brazilian Public Health System [12]. Additionally, a national survey in 2017 estimated 47,974 hip fractures in patients covered by the Public Health System [11]. If this is uplifted by 25% (to account for the private sector [24]), the number of hip fractures in Brazil (59,966) is markedly less than that estimated from data used to build the original FRAX model (80,640 for 2015) [6] but consistent with the present study (56,526). A comparison of probabilities of the updated model with the original model is given in Table 2 for men and women age 65 years with a prior fragility fracture together with other Latin American countries where a FRAX model is available. The probabilities of the updated model lay within the range observed in Latin America though the revision ranked somewhat lower.

**Table 2:** Ten-year probability of major osteoporotic fracture (MOF) and hip fracture (HF) in men and women age 65 years with a prior fragility fracture. (Body mass index was set to 25 g/m^2^; no BMD entered)

Country	Men		Women	
	MOF	HF	MOF	HF
Brazil (original)	4.1	1.2	7.1	2.0
Brazil (update)	3.4	0.8	5.9	1.4
Argentina	6.7	1.7	12	3.1
Ecuador	1.6	0.5	2.6	0.7
Chile	3.8	1.1	6.5	1.8
Colombia	4.1	1.1	7.1	1.8
Mexico	6.0	1.5	10	2.6
Venezuela	2.9	0.7	4.9	1.2

The BRAVOS study, on which the present report is based [13], confirms that there are large regional differences in the incidence of hip fracture in Brazil [11, 12]. Lowest rates are reported in the North and Northeast regions with progressively higher rates in the Centralwest, South and Southeast regions, respectively [12]. Regional differences of similar magnitude are reported elsewhere, and fracture rates are generally higher in urban communities than in rural communities as shown in Argentina [25], Croatia [26], Norway [27–29], Sweden [30, 31], Switzerland [32], Turkey [33] and in the USA [34, 35]. Reasons for these differences are conjectural but include differences in vitamin D status, everyday level of physical activity, factors related to socioeconomic prosperity and racial admixture [13, 31, 36, 37].

The regional differences have led to the view that more than one FRAX model is required for Brazil [12] as is available for the USA [38], Singapore [39], Malaysia [40] and South Africa [41]. The principal reason for the development of a single rather than regional model is the difficulty in the practical application of several regional models with uncertain boundaries. Moreover, the heterogeneity in incidence may be explained in part by differences in ethnic mix. There are, however, no studies available that characterise fracture risk by ethnicity in Brazil, though lower fracture rates are well established in blacks compared with whites in the USA and South Africa [41, 42]. Belem has a predominance of brown and blacks (72%), compared to whites (28%) whereas in Vitoria the prevalence ratio is 59% vs. 42%, respectively [13]. Assuming that race/ethnicity explains the difference in incidence between Belem and Vitoria then the incidence in Joinville (ratio 17% vs. 84%) would be 241/100,000 rather than the observed rate of 94/100,000. This suggests that race is unlikely to explain the totality of regional differences. Notwithstanding, future studies on the epidemiology of fracture would benefit by the characterisation of race.

The present study has several limitations. The data on hip fracture rates are based on regional rather than national estimates and are not necessarily representative of the whole country. The assumption is made that the cities reflect the regional epidemiology and that the three regions are representative of Brazil. Interestingly, the weighted ethnicity would approximate that of Brazil (White 49.3%, Brown 45% and Black 6.9%). Additionally, the reporting of conservative management or non-admission rates to hospital may have differed between regions introducing an unquantifiable bias. As noted above, not only may fracture rates differ according to ethnicity but so do death rates [43] that would in turn affect fracture probabilities. We also assumed that age- and sex-specific ratios of major osteoporotic fracture to hip fracture in different ethnic groups in Brazil were comparable, an assumption that could not be tested. In addition, the relative importance of the beta coefficients for death and fracture was assumed to be similar in Brazil as in the populations used to create and validate FRAX [44].

We conclude that age- and sex-specific hip fracture rates are significantly lower than previously reported in Brazil. A revision of the FRAX model for Brazil provides lower fracture probabilities than those derived from the original FRAX model for Brazil. Despite large differences between models, differences in the rank order of fracture probabilities were minimal.
